# Improves the *In Vitro* Developmental Competence and Reprogramming Efficiency of Cloned Bovine Embryos by Additional Complimentary Cytoplasm

**DOI:** 10.1089/cell.2018.0050

**Published:** 2019-02-07

**Authors:** Lianguang Xu, Ayman Mesalam, Kyeong-Lim Lee, Seok-Hwan Song, Imran Khan, M.M.R. Chowdhury, Wenfa Lv, Il-Keun Kong

**Affiliations:** ^1^ Division of Applied Life Science (BK21 Plus), Gyeongsang National University, Jinju, Republic of Korea.; ^2^ Department of Theriogenology, Faculty of Veterinary Medicine, Zagazig University, Zagazig, Egypt.; ^3^ Animal Genetic Resources Research Center, National Institute of Animal Science, RDA, Namwon, Republic of Korea.; ^4^ Division of Animal Reproduction and Breeding, Department of Animal Science, Jilin Agricultural University, Changchun, Republic of China.; ^5^ Institute of Agriculture and Life Science, Gyeongsang National University, Jinju, Republic of Korea.

**Keywords:** somatic cell nuclear transfer, cytoplasm injection cloning technology, bovine, embryo quality, reprogramming efficiency

## Abstract

Somatic cell nuclear transfer (SCNT) is a useful technology; however, its efficiency is low. In this study, we investigated the effects of cytoplasmic transfer into enucleated oocytes on the developmental competence and quality of cloned preimplantation bovine embryos via terminal deoxynucleotidyl transferase dUTP nick-end labeling, quantitative reverse transcription PCR, and immunocytochemistry. We used cytoplasm injection cloning technology (CICT), a new technique via which the cytoplasmic volume of an enucleated oocyte could be restored by injecting ∼30% of the cytoplasm of a donor oocyte. The percentages of embryos that underwent cleavage and formed a blastocyst were significantly higher (*p* < 0.05) in the CICT group than in the SCNT group (28.9 ± 0.8% vs. 20.2 ± 1.3%, respectively). Furthermore, the total cell number per day 8 blastocyst was significantly higher in the CICT group than in the SCNT group (176.2 ± 6.5 vs. 119.3 ± 7.7, *p* < 0.05). Moreover, CICT increased mitochondrial activity, as detected using MitoTracker^®^ Green. The mRNA levels of DNA methyltransferase 1 and DNA methyltransferase 3a were significantly lower (*p* < 0.05) in the CICT group than in the SCNT group. The mRNA level of DNA methyltransferase 3b was lower in the CICT group than in the SCNT group; however, this difference was not significant (*p* > 0.05). Taken together, these data suggest that CICT improves the *in vitro* developmental competence and quality of cloned bovine embryos.

## Introduction

Reconstructed embryos can be generated by injecting a highly differentiated somatic cell nucleus into an enucleated oocyte. Although more than 20 mammalian species have been successfully cloned via somatic cell nuclear transfer (SCNT), this technique is inefficient (Su et al., 2015). For several species, only 0%–5% of reconstructed embryos develop into viable offspring (Solter, 2000; Wilmut et al., 2002; Young, 2003), and development is poorer, the number of cells per blastocyst is lower, and the incidence of apoptosis is higher than in embryos produced *in vivo* or *in vitro* (Thompson, 1997; Vajta and Gjerris, 2006; Wang et al., 1999).

The low efficiency of SCNT is likely due to incomplete reprogramming of the donor nucleus, and epigenetic defects are thought to underlie most of the developmental problems of these embryos (Ng and Gurdon, 2005; Vajta and Gjerris, 2006). Successful cloning is considered to be dependent on reprogramming of differentiated somatic cells into a totipotent embryonic-like state (Dean et al., 2001; Latham, 2005; Tian, 2004).

Although reprogramming of somatic donor cells is largely complete (Smith et al., 2005) and SCNT with these cells can give rise to healthy cloned animals (Campbell et al., 2005; Vajta and Gjerris, 2006), much evidence suggests that insufficient or aberrant reprogramming at random loci in the somatic genome can contribute to the abnormal expression of genes crucial for development and cause abnormalities in cloned animals (Dean et al., 2001; Latham, 2005; Tian, 2004).

Efforts have been made to improve somatic cell nuclear reprogramming by reducing methylation levels in the somatic genome (Adams et al., 2005; Enright et al., 2003; Sanfins et al., 2004) and increasing chromosome accessibility by suppressing histone deacetylase activity (Beyhan et al., 2007; Enright et al., 2003; Kishigami et al., 2006; Rybouchkin et al., 2006). The developmental competence of cloned embryos inversely correlates with the level of misregulation of these genes. In addition to the epigenetic state of donor cells, the quality of recipient oocytes may also influence the reprogramming efficiency (Hua et al., 2011; Ju and Rui, 2012; Zhou et al., 2009).

To improve the efficiency of bovine cloning, high-quality embryos must be produced. There have been few studies on the influence of the remaining cytoplasm in enucleated oocytes on the development competency of nuclear transfer embryos. It has been reported that nucleocytoplasmic ratio may be important in early mammalian embryo development (Peura et al., 1998). As cytoplasmic volume does not increase during the first few rounds of embryonic cell division, increasing the embryonic cytoplasm may help in increasing the total cell number in developing blastocyst (Ribeiro et al., 2009).

Some studies suggested that embryo aggregation is a promising means to improve both the blastocyst development rate and the quality of cloned bovine embryos (Misica-Turner et al., 2007; Tang and West, 2000). On the contrary, previous studies have reported that the cell numbers in the resulting blastocysts were significantly lower when 50% of the cytoplasm was removed from enucleated bovine oocytes (Peura et al., 1998).

Moreover, aggregated cloned mouse embryos have a higher total cell number than single, nonaggregated cloned embryos, although their development rate is not improved (Boiani et al., 2003). Cytoplasmic donation is a recently developed technique to increase the quality of recipient oocytes by injecting a fraction of cytoplasm from a donor oocyte before fertilization (Dale et al., 2001). In the present study, we injected around 30% of the cytoplasm of a donor oocyte into an enucleated recipient oocyte to restore its cytoplasmic volume and investigated the beneficial effects on the development of cloned bovine embryos *in vitro*.

## Materials and Methods

### Ethics statement

The Institutional Animal Care and Use Committee of the division of applied life sciences, department of animal science at Gyeongsang National University, Republic of Korea approved (Approval ID: GAR-110502-X0017) all the methods and experimental procedures in this study.

### Chemicals

Unless otherwise noted, all chemicals and reagents were obtained from Sigma-Aldrich (St. Louis, MO).

### Donor cell preparation

Donor somatic cells were derived from the skin tissue of Hanwoo cattle (Korean Native Cattle). In brief, skin tissue was washed three times with Dulbecco's phosphate-buffered saline (D-PBS; Invitrogen, Carlsbad, CA), finely cut into 1 mm^2^ pieces, and digested in 0.25% (v/v) Trypsin-ethylenediaminetetraacetic acid solution (Gibco BRL; Life Technologies, Grand Island, NY) at 37°C for 1 hour. Thereafter, cells were washed three times with donor cell culture medium (Dulbecco's modified Eagle's medium [DMEM; Gibco] supplemented with 15% [v/v] fetal bovine serum [FBS; Gibco], 1% [v/v] l-glutamine, 1% [v/v] nonessential amino acids, and 1% [v/v] penicillin–streptomycin [P/S]), centrifuged at 1000 rpm for 2 minutes, and seeded into a 100 mm plastic dish (Becton Dickinson, Franklin Lakes, NJ).

Seeded cells were subsequently cultured in donor cell culture medium at 37°C in a humidified atmosphere of air containing 5% CO_2_ for 10–14 days. Cells at passage 3 were frozen in DMEM supplemented with 10% (v/v) FBS and 10% (v/v) dimethyl sulfoxide and stored in liquid nitrogen. Cells were thawed, cultured until passage 4–8, until they became confluent and used for cloning.

### Oocyte collection and in vitro maturation

*In vitro* maturation (IVM) was performed as previously described (Mesalam et al., 2018). Ovaries of Hanwoo cattle were obtained from a local abattoir and transported to the laboratory within 2 hours in sterile saline at 35°C. Cumulus-oocyte complexes (COCs) were aspirated from follicles with a diameter of 2–8 mm using an 18-gauge needle attached to a vacuum pump.

COCs with evenly granulated cytoplasm and more than three layers of compacted cumulus cells were selected and washed in Tyrode lactate-HEPES (TL-HEPES) medium (114 mM sodium chloride, 3.2 mM potassium chloride, 2 mM sodium bicarbonate, 0.34 mM sodium biphosphate, 10 mM sodium lactate, 0.5 mM magnesium chloride, 2 mM calcium chloride, 10 mM HEPES, 1 μL/mL phenol red, and 1% [v/v] P/S) and then in IVM medium (TCM-199 [Gibco] supplemented with 10% [v/v] FBS, 1 μg/mL estradiol-17β, 10 μg/mL follicle-stimulating hormone, 0.6 mM cysteine, and 0.2 mM Na-pyruvate), transferred to a four-well dish (Thermo Fisher Scientific, Waltham, MA) containing 600 μL of IVM medium, and incubated in a humidified atmosphere of air containing 5% CO_2_ at 38.5°C for 22–24 hours.

### *In vitro* fertilization

Matured COCs were fertilized as previously described (Mesalam et al., 2018). Semen was thawed in a water bath at 37°C for 1 minute. Sperms were then washed and pelleted in D-PBS by centrifugation at 1800 rpm for 5 minutes at room temperature. The pellet was carefully resuspended in *in vitro* fertilization (IVF) medium (Tyrode's lactate solution supplemented with 6 mg/mL bovine serum albumin [BSA], 22 μg/mL sodium pyruvate, 100 IU/mL penicillin, and 0.1 mg/mL streptomycin) containing 20 μg/mL heparin and incubated in a humidified atmosphere of air containing 5% CO_2_ at 38.5°C for 15 minutes to facilitate capacitation.

The sperm suspension was diluted in IVF medium (final density of 1–2 × 10^6^ sperm/mL). Matured COCs were transferred to four-well dishes containing sperm in 600 μL of IVF medium and then incubated in a humidified atmosphere of air containing 5% CO_2_ at 38.5°C for 18–20 hours.

### Nuclear transfer

After 22–24 hours of culture in IVM medium, cumulus cells were stripped from COCs by repeated pipetting in 0.1% (v/v) bovine testicular hyaluronidase prepared in TL-HEPES. Denuded oocytes with a first polar body were selected for enucleation, which was conducted as previously described (Lee et al., 2010). In brief, enucleation was achieved by aspirating the first polar body and a small amount of the surrounding cytoplasm in a droplet of TCM-199 media supplemented with 7.5 μg/mL cytochalasin B (CB) and 0.3% BSA. Donor somatic cells were immersed in Sendai virus (SV; Cosmo Bio, Tokyo, Japan) solution for 1 minute as described previously (Song et al., 2011). In brief, freeze-dried inactivated SV envelope was combined with 260 μL suspension buffer then diluted 1:4 (v/v) with fusion buffer.

**FIG. 1.**
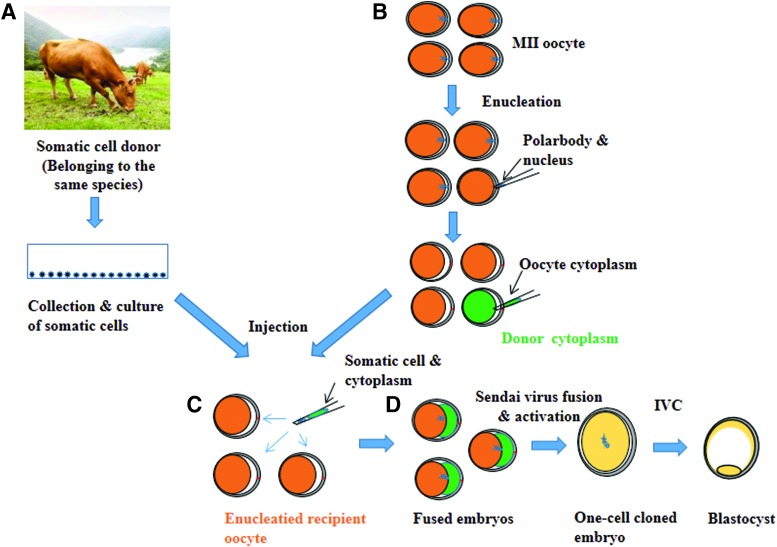
Schematic diagram of CICT. **(A)** Skin cells were collected from a donor cow and cultured *in vitro*. **(B)** Matured oocytes were enucleated. **(C)** The donor cell and oocyte cytoplasm (*green*) were injected into the enucleated recipient oocyte (*orange*). **(D)** The reconstructed oocytes were fused, and presumed cloned embryos were allowed to develop to the blastocyst stage *in vitro*. CICT, cytoplasm injection cloning technology. Color images available online.

Thereafter, a single-round donor somatic cell (<20 μm) was injected into the perivitelline space of each enucleated oocyte using a manipulation pipette (SCNT group). Alternatively, around 30% of cytoplasm from a donor oocyte was codelivered with somatic cell to the enucleated recipient oocyte to restore the cytoplasmic volume (cytoplasm injection cloning technology [CICT] group, Fig. 1). The reconstructed oocytes were fused via the SV-mediated fusion method (Song et al., 2011) and then incubated in SOF + BSA + ITS + EGF medium (Mesalam et al., 2018) supplemented with 5 μg/mL CB for 2 hours. Successfully reconstructed oocytes were activated by incubation in 5 μM ionomycin for 5 minutes, followed by incubation in 2 mM 6-dimethylaminopurine for 4 hours in a humidified atmosphere of air containing 5% CO_2_ at 38.5°C.

### *In vitro* culture

After incubation with sperm for 20 hours and/or activation of reconstructed oocytes, presumed zygotes/activated embryos were washed extensively and cultured in 80 μL droplets of SOF + BSA + ITS + EGF medium (20 embryos per droplet) covered with mineral oil in a humidified atmosphere of air containing 5% CO_2_ at 38.5°C. Half the culture medium was replenished every 2 days. Cleavage of reconstructed embryos and *in vitro* fertilized embryos was checked at day 2 after fusion and day 3 after IVF, respectively (day 0 = day of IVF or fusion).

The blastocyst developmental competence was monitored under a stereomicroscope after 8 days of culture. Day 8 blastocysts were washed three times in TL-HEPES, transferred to fixative (4% [v/v] paraformaldehyde prepared in 1 M phosphate-buffered saline [PBS]), and stored at 4°C until apoptotic and total cells were analyzed. For gene expression analysis, day 8 blastocysts were transferred to a 1.5 mL Eppendorf tube, snap-frozen in liquid nitrogen, and stored at −80°C.

### Terminal deoxynucleotidyl transferase dUTP nick-end labeling

The numbers of total and apoptotic cells in day 8 blastocysts were determined by terminal deoxynucleotidyl transferase dUTP nick-end labeling (TUNEL) as previously described (Mesalam et al., 2017a). TUNEL was performed using an In Situ Cell Death Detection Kit (Fluorescein; Roche Diagnostics Corp., Indianapolis, IN) according to the manufacturer's protocol. In brief, day 8 blastocysts were washed three times in TL-HEPES, transferred to fixative (4% [v/v] paraformaldehyde prepared in 1 M PBS), fixed embryos were washed in 0.3% (w/v) polyvinylpyrrolidone (PVP) prepared in 1 M PBS (PVP-PBS) before being incubated in permeabilization solution (0.5% [v/v] Triton X-100 and 0.1% [w/v] sodium citrate) at room temperature for 30 minutes.

After permeabilization, the embryos were washed twice in PVP-PBS and incubated with fluorescein-conjugated deoxyuridine triphosphate and terminal deoxynucleotide transferase in the dark for 1 hour. TUNEL-stained embryos were then washed in PVP-PBS, incubated in PVP-PBS containing 10 μg/mL Hoechst 33342 for 10 minutes, washed twice in PVP-PBS to remove excess Hoechst 33342, and mounted on glass slides. The number of cells per blastocyst was counted using an epifluorescence microscope (Olympus IX71, Tokyo, Japan) equipped with a mercury lamp. TUNEL-positive cells appeared bright red, indicating the occurrence of apoptosis. The TUNEL assay was performed three times. A total of 15 blastocysts were analyzed per group.

### Assessment of mitochondrial activity

Mitochondrial activity was analyzed using a commercial kit (MitoTracker^®^ Green FM; Invitrogen) according to the manufacturer's instructions. In brief, fixed day 8 blastocysts were washed three times with D-PBS and incubated in 125 nM MitoTracker Green FM at 37°C for 30 minutes (Mesalam et al., 2017b). Then, blastocysts were rinsed twice with D-PBS and labeled with Hoechst 33342 in the dark at room temperature for 10 minutes.

After staining, blastocysts were placed on a glass slide and examined under a confocal laser-scanning Olympus FluoView FV1000 microscope. The excitation wavelength was 594 nm, and emission was read at 608 nm. Mitochondrial fluorescence was quantified using ImageJ (National Institutes of Health, Bethesda, MD; https://imagej.nih.gov/ij) after normalization via subtracting the background intensity from each image. Experiments were repeated three times, with 20 blastocysts examined per group.

### Mitochondrial characterization

To track the distribution of donor cytoplasm after injection of recipient enucleated oocytes, we performed mitochondrial staining of donor cytoplasm using MitoTracker Green FM. We collected different stage embryos (directly, 2 or 6 hours after injection and cleaved embryos) and labeled with Hoechst 33342 in the dark at room temperature for 10 minutes. After staining, different stage embryos were placed on a glass slide and examined under a confocal laser-scanning Olympus FluoView FV1000 microscope.

### mRNA extraction and complementary DNA reverse transcription

Day 8 blastocysts were transferred to a 1.5 mL Eppendorf tube, snap-frozen in liquid nitrogen, and stored at −80°C. Total RNA was extracted from four biological replicates, with five blastocysts per replicate (day 8, *n* = 20 per group), using an Arcturus PicoPure RNA Isolation Kit (Life Technologies, Inc., Foster City, CA) according to the manufacturer's guidelines. The concentration and purity of RNA were determined using a NANO DROP 2000c instrument (Thermo Fisher Scientific, Wilmington, DE). RNA samples were stored at −80°C until use. mRNA was reverse-transcribed into first-strand complementary DNA (cDNA) using an iScript™ cDNA Synthesis Kit (Bio-Rad Laboratories, Hercules, CA) according to the manufacturer's instructions. Finally, cDNA was stored at −80°C until used for quantitative reverse transcription PCR (RT-qPCR).

### RT-qPCR analysis

Gene-specific primers were designed using Primer3Plus software (http://primer3plus.com/cgi-bin/dev/primer3plus.cgi) and are presented in Table 1. RT-qPCR analysis was performed using a CFX98 real-time system (Bio-Rad Laboratories, Inc.) with a reaction volume of 10 μL containing 0.2 mM of each bovine-specific primer, 1 × iQ SYBR Green Supermix (iQ SYBR Green Supermix kit; Bio-Rad Laboratories, Inc.), and 3 μL of diluted cDNA. All cDNA samples were subjected to analysis using glyceraldehyde-3-phosphate dehydrogenase (*GAPDH*)-specific primers to detect any variation in expression of this internal control gene.

**Table 1.** Primer Sequences for Quantitative Reverse Transcription PCR

*Gene*	*Primer sequence*	*Accession number*	*Product size (bp)*
*DNMT1*	F: AGGGAGACGTGGAGATGCTG	AY244709	194
	R: CATGGAGCGCTTGAAGGAG		
*DNMT3a*	F: AGACATGTGGGTTGAACCCG	AY271298	188
	R: GGCTCCCACAAGAGATGCAG		
*DNMT3b*	F: CAGGATGGGAAGGAGTTTGGA	AY244710	151
	R: CACCAAACCACTGGACCCAC		
*GAPDH*	F: CCCAGAATATCATCCCTGCT	NM_001034034	185
	R: CTGCTTCACCACCTTCTTGA		

*GAPDH*, glyceraldehyde-3-phosphate dehydrogenase; *DNMT1*, DNA methyltransferase 1; *DNMT3a*, DNA methyltransferase 3a; *DNMT3b*, DNA methyltransferase 3b; F, forward; R, reverse.

After confirming that relative *GAPDH* expression did not significantly differ among the samples, all transcripts were quantified in independent analyses. PCR involved a denaturation step (95°C for 3 minutes) followed by 44 cycles of 95°C for 15 seconds, 57°C for 20 seconds, and 72°C for 30 seconds, and a final extension at 72°C for 5 minutes. Amplification was followed by melting curve analysis using progressive denaturation, in which the temperature was increased from 65°C to 95°C at a rate of 0.2°C/s, during which fluorescence was measured continuously. Quantitative analysis was performed using the ΔΔC(t) method. For all genes profiled, the intra- and interassay coefficients of variation were calculated using the following formula: standard deviation/mean × 100.

### Statistical analysis

Data are expressed as means ± standard errors of the mean and analyzed by a one-way analysis of variance using SPSS 18.0 (SPSS, Inc., Chicago, IL). Duncan's multiple range test was used to compare the groups. *p* < 0.05 was considered significant.

## Results

### Development of cloned preimplantation bovine embryos

We examined the effects of cytoplasmic transfer on cleavage of cloned preimplantation bovine embryos at day 2 and their blastocyst developmental competence at day 8. The fusion rate was significantly higher (*p* < 0.05) in the CICT group than in the SCNT group (82.0 ± 0.3% vs. 68.3 ± 1.5%; Table 2). Moreover, the percentage of 8–16 cell stage embryos in the CICT group was significantly higher (*p* < 0.05) than that in the SCNT group (61.5 ± 1.3% vs. 39.7 ± 2.1%), but lower than that in the IVF group (75.4 ± 1.3%) (Table 2). The percentage of embryos that developed to the blastocyst stage was significantly higher (*p* < 0.05) in the CICT group than in the SCNT group (28.9 ± 0.8% vs. 20.2 ± 1.3%) (Table 2).

**Table 2.** *In Vitro* Development of Embryos Cloned Using Different Methods

*Group*	*No. of cultured zygotes*	*No. of cloned oocytes*	No. (%) of fused embryos^*^	No. (%) ≥2–4 cell embryos^**^	No. (%) ≥8–16 cell embryos^**^	No. (%) of blastocysts^***^
IVF	258	—	—	219 (85.0 ± 1.5)^b^	194 (75.4 ± 1.3)^a^	80 (31.1 ± 1.1)^a^
SCNT	—	558	381 (68.3 ± 1.5)^a^	303 (79.5 ± 1.4)^a^	148 (39.7 ± 2.1)^b^	74 (20.2 ± 1.3)^b^
CICT	—	296	243 (82.0 ± 0.3)^b^	200 (82.0 ± 1.7)^ab^	149 (61.5 ± 1.3)^c^	70 (28.9 ± 0.8)^a^

Mean ± standard error of the mean.

^a–c^ Values with different superscripts in the same column are significantly different (*p* < 0.05).

^*^ Fusion rates were calculated based on the number of injected oocytes.

^**^ Cleavage rates were calculated based on the number of fused embryos for SCNT, CICT and based on the number of fertilized zygote for IVF.

^***^ Blastocyst development rates were calculated based on the number of fused embryos for SCNT and CICT and based on the number of presumed zygote for IVF.

SCNT, somatic cell nuclear transfer; CICT, cytoplasm injection cloning technology; IVF, *in vitro* fertilization.

### Quality of bovine blastocysts

**FIG. 2.**
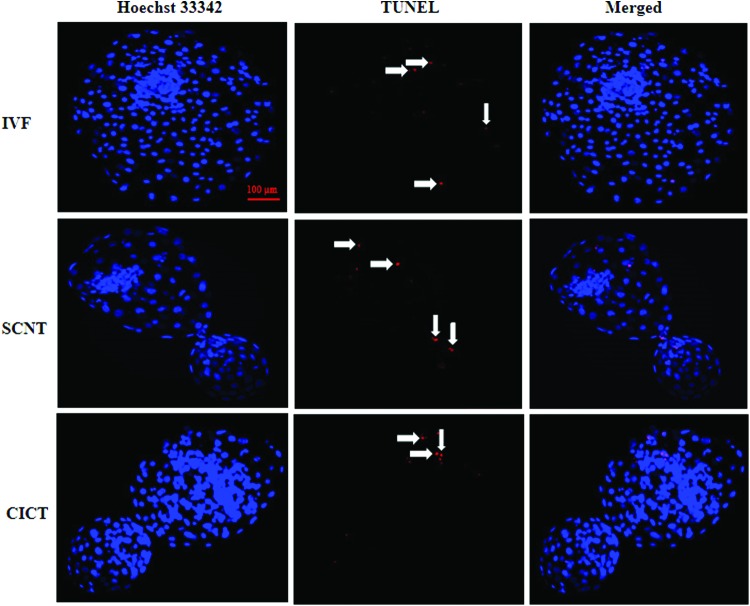
TUNEL of day 8 blastocysts in the IVF, SCNT, and CICT groups. Apoptotic cells were detected by TUNEL (*red*). Each sample was counterstained with Hoechst 33342 to visualize cell nuclei (*blue*). *White arrows* indicate apoptotic cells. Scale bar = 100 μm. TUNEL, terminal deoxynucleotidyl transferase dUTP nick-end labeling. SCNT, somatic cell nuclear transfer; IVF, *in vitro* fertilization. Color images available online.

To investigate whether CICT improved the quality of blastocysts, the numbers of total and apoptotic cells were determined in day 8 blastocysts. The total number of cells per blastocyst in the CICT group was significantly higher (*p* < 0.05) than that in the SCNT group (176.2 ± 6.5 vs. 119.3 ± 7.7), but was similar (*p* > 0.05) to that in the IVF group (184.1 ± 8.7) (Table 3; Fig. 2). By contrast, the number of apoptotic cells per blastocyst was not significantly different among three groups (4.4 ± 0.2, 4.1 ± 0.3 and 3.5 ± 0.4) (Table 3).

**Table 3.** Characterization of Day 8 Blastocysts

*Group*	*No. of blastocysts*	*No. of total cells per blastocyst*	*No. of apoptotic cells per blastocyst*
IVF	15	184.1 ± 8.7^a^	4.4 ± 0.2^a^
SCNT	15	119.3 ± 7.7^b^	4.1 ± 0.3^a^
CICT	15	176.2 ± 6.5^a^	3.5 ± 0.4^a^

^a,b^ Values with different superscript in the same columns are significantly different (*p* < 0.05).

### Mitochondrial activity

**FIG. 3.**
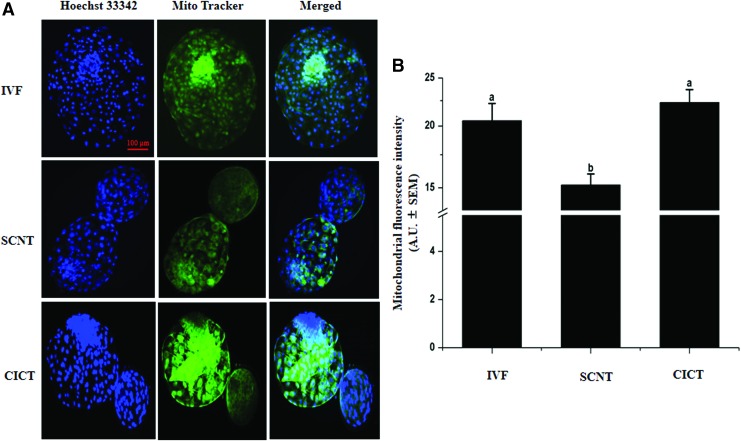
Fluorescence intensity of mitochondrial staining in day 8 blastocysts. **(A)** MitoTracker^®^ Green FM was used to label mitochondria (*green*). Nuclei were labeled with Hoechst 33342 (*blue*). Scale bar = 100 μm. **(B)** Fluorescence intensity of mitochondrial staining per blastocyst. *Columns* with different superscripts are significantly different (*p* < 0.05). Fluorescence intensities are expressed as arbitrary units ± standard errors of the mean. Color images available online.

We examined the effect of cytoplasmic transfer on the mitochondrial fluorescence intensity using MitoTracker Green FM. The fluorescence intensity of mitochondrial staining was significantly higher (*p* < 0.05) in the CICT group than in the SCNT group (22.3 ± 1.4 vs. 15.2 ± 0.8 arbitrary units); however, it did not significantly differ (*p* > 0.05) between the CICT and IVF groups (22.3 ± 1.4 vs. 20.5 ± 1.7 arbitrary units) (Fig. 3).

### Mitochondrial characterization

**FIG. 4.**
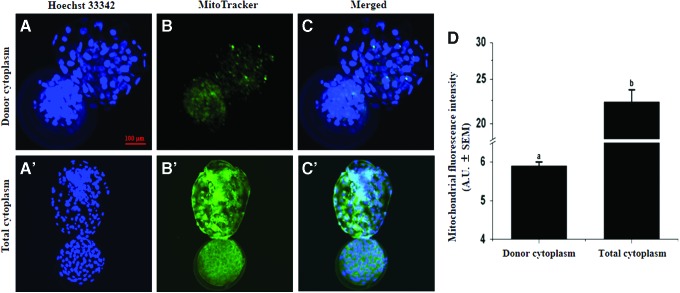
Fluorescence intensity of mitochondrial staining in CICT blastocysts. MitoTracker Green FM staining of donor cytoplasm **(A**–**C)** and total cytoplasm **(A′**–**C′)** in CICT group. Scale bar = 100 μm. **(D)** Mean values of the integrated optical density of mitochondrial staining per blastocyst. Columns with different superscripts are significantly different (*p* < 0.01). Color images available online.

We investigated the distribution of donor cytoplasm and the percentage of mitochondrial heterogeneity after CICT. Our results showed that the donor cytoplasm regularly distribute in the recipient oocyte after injection as well as different stages embryo (Supplementary Figs. S1 and S2; Supplementary Data are available online at www.liebertpub.com/cell). Moreover, the fluorescence intensity of donor mitochondria in day 8 blastocysts was 5.9 ± 0.1 arbitrary units, representing only 26.3% of total fluorescence intensity of day 8 blastocysts mitochondria (22.3 ± 1.4 arbitrary units) (Fig. 4).

### RT-qPCR analysis

**FIG. 5.**
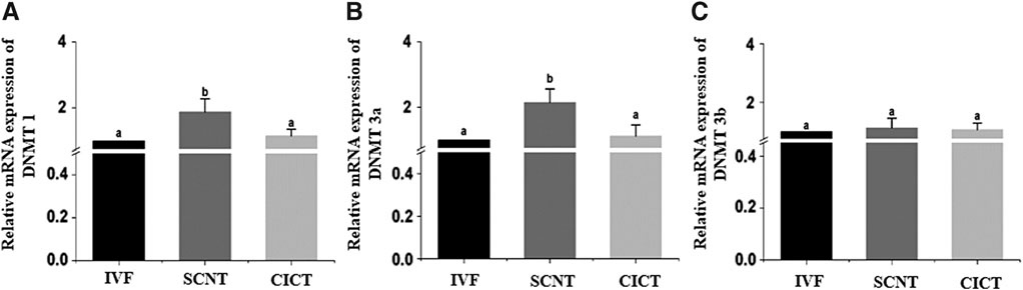
Relative mRNA expression levels of *DNMT* genes in blastocysts determined by quantitative reverse transcription PCR. Relative mRNA expression levels of *DNMT1* **(A)**, *DNMT3a* **(B)**, and *DNMT3b* **(C)** in blastocysts from the IVF, SCNT, and CICT groups. *Columns* with different superscripts are significantly different (*p* < 0.05). DNMT1, DNA methyltransferase 1; DNMT3a, DNA methyltransferase 3a; DNMT3b, DNA methyltransferase 3b.

RT-qPCR was performed to quantify the mRNA expression levels of DNA methyltransferase 1 (*DNMT1*), DNA methyltransferase 3a (*DNMT3a*), and DNA methyltransferase 3b (*DNMT3b*). The expression levels of these genes were normalized to that of the housekeeping gene *GAPDH*. The mRNA levels of *DNMT1* and *DNMT3a* were significantly lower (*p* < 0.05) in the CICT group than in the SCNT group; however, they did not significantly differ between the CICT and IVF groups (Fig. 5). Moreover, the mRNA level of *DNMT3b* was not significantly different among the three groups.

## Discussion

After the birth of Dolly in 1997, SCNT technique gained interest and several animal species has been produced by same technique (Verma et al., 2015). To overcome the limitations of SCNT, handmade cloning, also known as zona-free cloning or hand-guided technique, has been developed (Vajta et al., 2003). However, difficulty in obtaining newborns from zona-free embryos has been reported because zona-free embryos might be affected by toxic substances in culture media (Verma et al., 2015). In this study, we reported a new cloning technique, CICT, which can restore the cytoplasmic volume of enucleated oocyte without removing the zona pellucid.

High-quality embryos are required for successful animal cloning via SCNT (Tian et al., 2003). Various properties determine embryo quality, such as cell number, embryo morphology, gene expression, timing of development, mitochondrial activity, and DNA methylation (Akagi et al., 2011; Galli et al., 2002; Gambini et al., 2012). It has been reported that production of high-quality bovine SCNT blastocysts are highly related to the cytoplasmic volume (Peura et al., 1998) and fusion method (Song et al., 2011). Moreover, SV-mediated fusion improves the developmental competence and quality of bovine SCNT blastocysts (Song et al., 2011). In this study, we investigated the effects of cytoplasmic transfer into enucleated oocytes on the developmental competence and quality of cloned preimplantation bovine embryos.

We sought to improve the quality of cloned embryos using a new technique called CICT, which restored the cytoplasmic volume of an enucleated recipient oocyte by injecting around 30% of the cytoplasm of a donor oocyte. Embryo quality was evaluated by assessing the total cell number, mitochondrial activity, and gene expression. Fusion, cleavage (8–16 cell), and blastocyst formation rates were significantly higher in the CICT group than in the SCNT group, suggesting that restoration of the cytoplasmic volume in enucleated recipient oocytes enhanced the fusion rate by increasing the possibility of attachment between somatic cell and cytoplasm and subsequently improve the developmental competence and quality of cloned bovine embryos. Our results supported by previous study reported that increase in cytoplasmic volume had a positive effect on embryo development (Ribeiro et al., 2009).

The cell number per blastocyst has been used as an indicator of embryo quality (Zhu et al., 2014). The total cell number per SCNT embryo determines their viability after transfer (Buemo et al., 2016; Gambini et al., 2014). In several species, a low number of cells per cloned embryo are associated with a low survival rate after transfer (Das et al., 2003; Min et al., 2015; Rybouchkin et al., 2002). The total cell number per day 8 blastocyst was significantly higher in the CICT group than in the SCNT group, indicating that CICT can improve the quality of cloned embryos.

Mitochondria play key roles in many cell functions, such as calcium regulation, programmed cell death, and ATP production (Giorgi et al., 2012). Mitochondrial activity and sufficient ATP production are indicative of the high quality of cells and embryos (Van Blerkom, 2009; Van Blerkom et al., 1995).

Therefore, the mitochondrial fluorescence intensity could be potentially used to select good-quality embryos and thereby improve the efficiency of animal cloning (Verma et al., 2015). The mitochondrial fluorescence intensity was significantly higher in the CICT group, in which the total cell number per blastocyst was increased, than in the SCNT group. This is in agreement with our previous finding that the mitochondrial fluorescence intensity is increased in good-quality bovine embryos with a low number of apoptotic cells and a high number of total cells (Fakruzzaman et al., 2013; Ghanem et al., 2014).

The integrity and survival of all organisms are dependent on controlled gene expression and cell proliferation. During development of a fertilized embryo into a multicellular organism, cell-fate decisions are taken, and cell lineage- or tissue-specific gene expression patterns must be established and maintained (Lagger et al., 2002). DNA methylation modifies and regulates the chromatin structure and plays a crucial role in sustaining genomic stability and activating or suppressing gene expression (Jin et al., 2011; Lan et al., 2010). Cloned bovine embryos tend to have aberrant methylation patterns compared with *in vitro* fertilized embryos, indicative of inefficient reprogramming (Couldrey and Lee, 2010; Seisenberger et al., 2013).

Consistently, preimplantation SCNT embryos display marked differences in gene expression, which affects their developmental competence after implantation (Jang et al., 2005). DNA methyltransferases play important roles in the regulation of gene transcription via chromatin remodeling (Jin et al., 2011). Moreover, DNA methylation is mediated by DNA methyltransferases, such as Dnmt1, Dnmt3a, and Dnmt3b, which are considered significant barriers to reprogramming (Wang et al., 2017). Gene expression of *DNMT1*, *DNMT3a*, and *DNMT3b* correlates with the reprogramming efficiency of cloned embryos (Ha et al., 2015; Sawai et al., 2010). In this study, we investigated the expression profiles of genes related to chromatin remodeling. Expression of *DNMT1* and *DNMT3a* was significantly lower in the CICT group than in the SCNT group. This indicates that CICT can be used as a new cloning technology to improve epigenetic reprogramming during bovine cloning, however, this need further studies to test this hypothesis by embryo transfer in bovine species.

In conclusion, our results show that restoration of the cytoplasmic volume in enucleated recipient oocytes via CICT enhances the developmental competence and quality of cloned bovine embryos. This study might help researchers to mitigate the adverse effects associated with enucleation, such as a reduced cytoplasmic volume, and thereby improve the reprogramming efficiency of cloned bovine embryos.

## Supplementary Material

Supplemental data

Supplemental data
