# Supplementary material for: Improves the In Vitro Developmental Competence and Reprogramming Efficiency of Cloned Bovine Embryos by Additional Complimentary Cytoplasm
Source: Cell Reprogram. 2019 Feb 7;21(1):51–60. doi: 10.1089/cell.2018.0050 (PMC6383574; doi:10.1089/cell.2018.0050)
Supplement: Supplemental data [file Supp_Fig1.pdf]

Supplementary Data

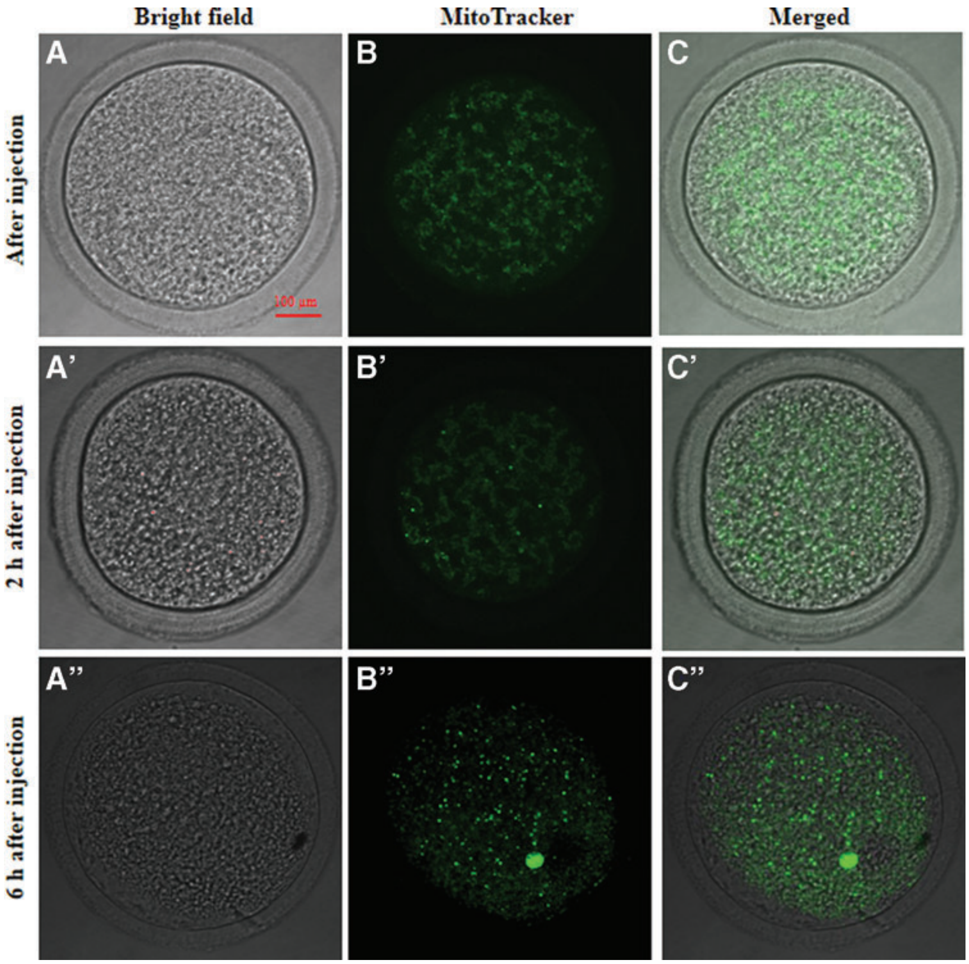

SUPPLEMENTARY FIG. S1. Fluorescence intensity of mitochondrial staining in different stages of CICT embryos. MitoTracker® Green staining of donor cytoplasm in the **(A–C)** recipient oocyte after injection, **(A′–C′)** 2 hours after injection, and **(A″–C″)** 6 hours after injection. CICT, cytoplasm injection cloning technology.
