# Supplementary material for: Improves the In Vitro Developmental Competence and Reprogramming Efficiency of Cloned Bovine Embryos by Additional Complimentary Cytoplasm
Source: Cell Reprogram. 2019 Feb 7;21(1):51–60. doi: 10.1089/cell.2018.0050 (PMC6383574; doi:10.1089/cell.2018.0050)
Supplement: Supplemental data [file Supp_Fig2.pdf]

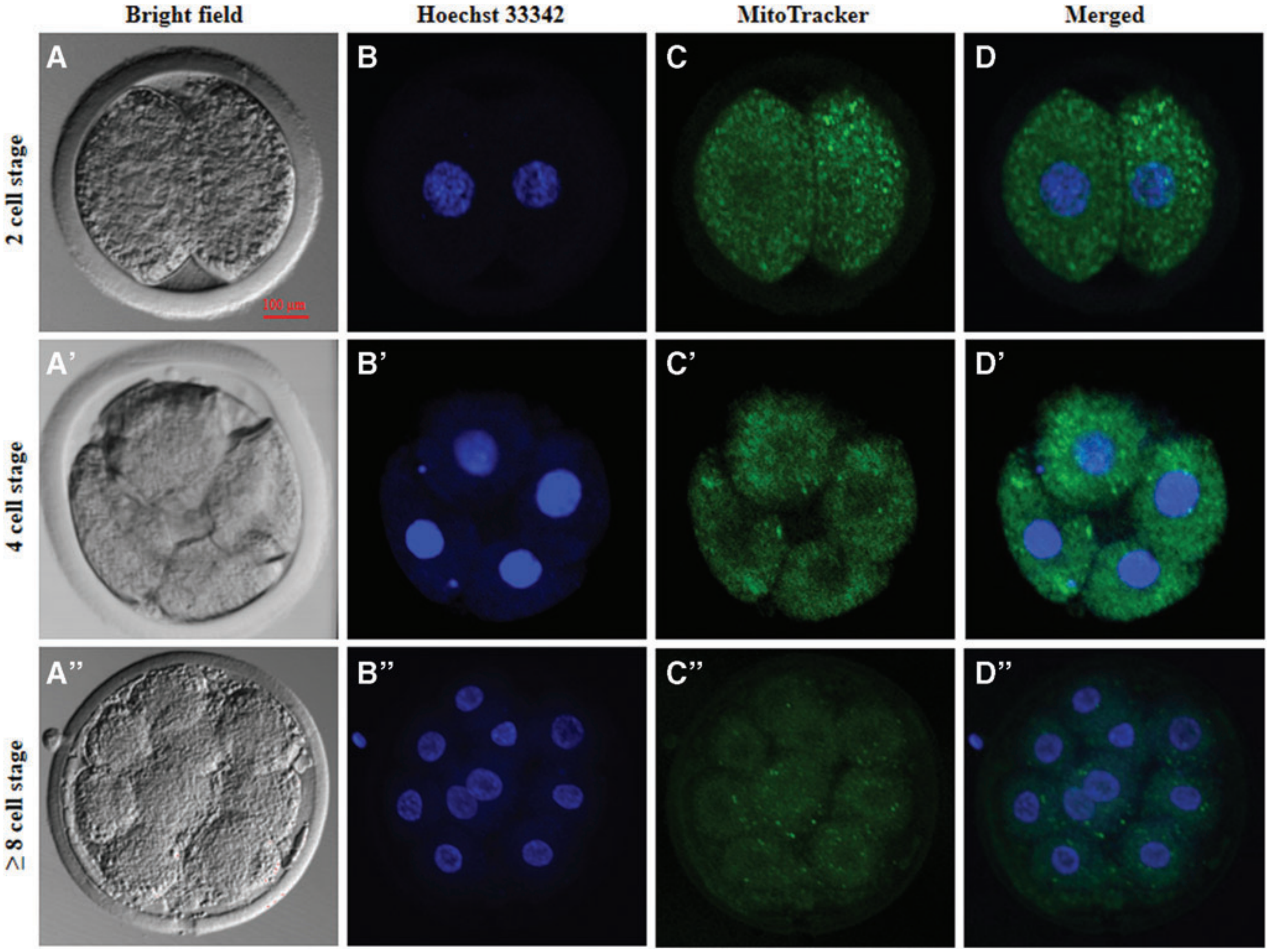

SUPPLEMENTARY FIG. S2. Fluorescence intensity of mitochondrial staining in different stages of CICT embryos. MitoTracker Green staining of donor cytoplasm in the **(A–D)** two-cell stage, **(A′–D′)** four-cell stage, and **(A″–D″)** ≥8-cell stage embryos.
